# When your PhD (almost) falls apart

**DOI:** 10.7554/eLife.81548

**Published:** 2022-07-11

**Authors:** Dominik Refardt

**Affiliations:** 1 Institute for Natural Resource Sciences, Zurich University of Applied Sciences Zurich Switzerland

**Keywords:** sparks of change, research culture, scientific mistakes, PhD

## Abstract

As a chance observation threatens to unravel several years of work, a PhD student must choose what to do next.

When I was ten, I nearly drowned in a river. Engulfed by water, about to be completely overwhelmed with panic, my rational self suddenly stepped out to ensure that at least some part of me remained calm. It seemed to work. I remember that I smiled while under water, gathered strength, and then shouted whenever I surfaced from the swirls until someone grasped my hand. A decade later, the same feeling came over me as I realised that what I was seeing down a microscope threatened to destroy my entire PhD.

I had handed in my thesis only a few moments before. Some great chapters, some mediocre ones, some published, all of them the proud result of four years of hard work. I just felt relief. Done! Finally! PhD, here I come! I expected this moment to be etched in my memory, but all I can vaguely recall is the shape of the desk on which I placed the manuscript. What happened a few minutes later, however, I remember vividly.

I immediately ventured back to the lab to conduct follow-up experiments and side projects. I took out some tissue samples and inspected them under the microscope; they were all negative controls and therefore supposed to be uninfected. Except they were not. I could see clear clusters of parasite spores, where none should have been. I reclined from the microscope; time stood still, and everything became quiet. Very slowly, it dawned on me what this meant for several of my experimental results, for my thesis, for my hard work in the last few years. A massive storm was about to hit me.

My research focused on the relationships between the water flea *Daphnia magna* and two species of parasites that live inside its gut and which are distantly related to fungi. I thought I had developed genetic markers for all the parasites in my experiments, yet now I could clearly see that one had escaped me. I would soon realise that, morphologically indistinguishable and genetically invisible, it had infected many of my samples, often hiding alongside one of my 'known' parasites. Worse, it was much more difficult to eradicate than other species and many of my ‘cured’ negative controls still harboured it.

As the pieces started to assemble, again the ‘rational me’ surfaced as if it was the only way I could sever ties with my emotions, disentangle from them before the meltdown. It seemed to know that, before long, a barrage of thoughts and feelings would take over, some of them catastrophic from a scientific point of view. Like simply not telling anyone and pretending that this had never happened.

Like a puppet I stood up, left the lab, and went straight to the office of my best friend in the group. I think I knew, deep down, that I would be able to confess to her. She was a postdoc but had never judged me on whether I asked clever questions in seminars, or if I already had papers. To her, I was just a friend, with the usual flaws and weaknesses. I told her everything at once. Now that this was out in the open, there would be no way to hide the mistake. I am grateful to have had someone like her around because things would have turned out very differently otherwise.

Next, I went to my supervisor’s office, told him what I just observed and that I needed my thesis back. I don’t remember his reaction; he probably was understanding, or at least remained calm — anything else would have left a mark. Later that day I met my brother for lunch, and he asked how I was. I heard myself say that nothing was OK, that I had probably wasted years of work, that I wasn’t sure I could graduate. The storm finally caught up with me, and I started to cry.

The next few days are lost in a haze, and I can’t remember how deep the hole I fell into was. I began to clean up the mess, sifting through data and notes, sorting what was still valid and what had to be trashed. But as I was going through years of lab notes, pieces of evidence started to connect.

During my PhD I had sometimes caught glimpses of another, peculiar inhabitant in the gut of *D. magna*, and I had become somewhat obsessed with it. I kept jars of live water fleas infected with it, but I could never manage to sequence it, even just partially. Once, I had also found a segment of a coding sequence across many unrelated samples; it resembled fungal DNA, and I had discarded it as probably belonging to one of the many commensal fungi living in the fleas.

However, I now realised that this intriguing bit of fungal DNA clearly occurred wherever I had co-infections with the mysterious parasite. In parallel, I recalled having once noted the location of the infection in the gut for all combinations of hosts and parasites. This helped me realize that the parasites I studied always occupied the upper part of the gut whereas my mystery bug preferred the lower end. I therefore knew exactly where it was hiding, and it matched where the ominous bit of fungal DNA appeared perfectly. My misery gave way to excitement. Hiding in plain sight, a creature was lurking which we knew nothing about. A new parasite! And not just another microsporidium, like the parasites I was studying. Sequence evidence showed it was very likely the missing link that would confirm that microsporidia had evolved from within fungi – a hypothesis that was the subject of intense debate at the time.

Armed with all this evidence, I went back to my supervisor and managed to convince him that I was onto something. I left the group shortly afterwards, but he and his students spared no effort in hunting down this new parasite. They were successful and I am proud to be a co-author of the resulting paper, which was well-received. Meanwhile, I cut out some results from my thesis, and resubmitted it. Eventually, I successfully defended my PhD, celebrating with a gifted self-made Matterhorn mountain of chocolate and by shaving all of my hair (a story for another time).

This was all years ago, and I have since recovered from the shock and found my place in science. What remains is the conviction that in research, slow and painstaking work almost always pays off — even though, as a group leader with administrative duties, I have now learned that some jobs are best done quick and dirty. I was only able to spot the parasite because I had meticulously recorded casual observations that looked insignificant at the time, and I was allowed to pursue my own questions no matter how naive they may appear then. Had I not been allowed to sequence my samples so thoroughly, for example, I would have never identified the unknown bug. Doing so must have looked like a huge waste of time but thank goodness nobody ever told me to stop.

Having my own students also made me realise how much trust my supervisor had put into me, paying for countless DNA extraction kits, Taq polymerases and sequencing jobs without ever complaining. Only now do I understand how difficult it is to give freedom to your students, to let them make mistakes, to trust them when their approach to a problem differs from yours. But I strive to be as generous with them as my supervisor was with me. We dare much more when we know we are allowed to fail.

## Share your experiences

This article is a Sparks of Change column, where people around the world share moments that illustrate how research culture is or should be changing. Have an interesting story to tell? See what we’re looking for and the best ways to get in touch here.

